# Carotid chemoreceptor denervation does not impair hypoxia-induced thermal downregulation but vitiates recovery from a hypothermic and hypometabolic state in mice

**DOI:** 10.1038/s41598-019-41546-x

**Published:** 2019-03-26

**Authors:** Sebastiaan D. Hemelrijk, Thomas M. van Gulik, Michal Heger

**Affiliations:** 1 Department of Surgery, Amsterdam University Medical Centers, location Academic Medical Center, University of Amsterdam, Amsterdam, The Netherlands; 2 Department of Intensive Care, Diakonessenhuis Utrecht, Utrecht, The Netherlands; 3 Department of Pharmaceutics, College of Medicine, Jiaxing University, Jiaxing, Zhejiang PR China

## Abstract

Induction of hypothermia and consequent hypometabolism by pharmacological downmodulation of the internal thermostat could be protective in various medical situations such as ischemia/reperfusion. Systemic hypoxia is a trigger of thermostat downregulation in some mammals, which is sensed though carotid chemoreceptors (carotid bodies, CBs). Using non-invasive thermographic imaging in mice, we demonstrated that surgical bilateral CB denervation does not hamper hypoxia-induced hypothermia. However, the recovery from a protective and reversible hypothermic state after restoration to normoxic conditions was impaired in CB-resected mice versus control animals. Therefore, the carotid chemoreceptors play an important role in the central regulation of hypoxia-driven hypothermia in mice, but only in the rewarming phase.

## Introduction

Hibernation is a hypometabolic state that manifests in several animal species, including mammals, to protect against environmental stressors such as cold and starvation^1,2^. Hibernators naturally engage hypometabolism during the winter period in response to altering day/night cycles, decreasing ambient temperature (*T*_a_), and shortage of food. Hypometabolic signaling comprises a downmodulation of the bodies’ thermostat (a circuit of thermosensitive neurons) in the preoptic anterior hypothalamus (POAH) via a process referred to as anapyrexia (i.e., POAH-controlled hypothermia). During anapyrexia, controlled deviation from the thermoneutral zone (Z_tn_), which is a tightly regulated body temperature (*T*_b_) range at which the organism functions optimally, results in a decrease in *T*_b_ to a state of (deep) hypothermia, and, in accordance with Arrhenius’ law, in a reduction of metabolism^2–4^. Correspondingly, a hypometabolic state concurs with a reduction in oxygen consumption (*V*O_2_) and carbon dioxide production due to stalled aerobic respiration.

Artificial induction of hibernation by an alleged hibernation induction trigger (HIT) has been a subject of biomedical research for decades^5^. By alleviating the harmful *V*O_2_:O_2_ supply (*D*O_2_)-mismatch that arises during several medical conditions (e.g., ischemia and reperfusion during surgical procedures or following acute vascular occlusion), the artificial induction of hibernation could enable better management of some of these contrived clinical procedures (e.g., vascular inflow occlusion during liver resection and transplantation)^6–11^.

**Figure 1:**
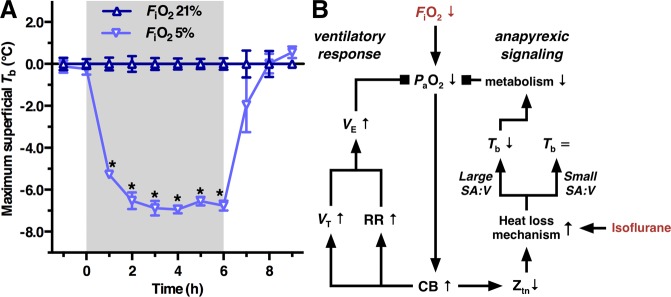
The anapyrexic effects of hypoxia: a role for the carotid chemoreceptors. (**A**) The hypothermic effects of 6-h hypoxia exposure (*F*_i_O_2_ = 5%) at a *T*_a_ of 21.3 ± 0.3 °C, presented as mean ± SEM maximum superficial *T*_b_ normalized to normoxia control values (*F*_i_O_2_ of 21%) in mice (adapted from Hemelrijk *et al*.^18^). (**B**) Schematic overview of hypoxia sensing mechanisms by the carotid body (CB). Consequent to a hypoxic challenge by exposure to a reduced *F*_i_O_2_, the partial arterial oxygen pressure (*P*_a_O_2_) decreases and the CBs are activated. CB activation is followed by the ventilatory response (left) as well as anapyrexic signaling (right). The hypoxic ventilatory response comprises predominately an increase in respiratory rate (RR) and, to a lesser extent, an increase in tidal volume (*V*_T_), both contributing to an increase in minute volume (*V*_E_). Anapyrexic signaling is activated by downmodulation of the thermoneutral zone (Z_tn_), which promotes a heat loss mechanism and causes a reduction in body temperature (*T*_b_) if the surface area (*SA*):volume (*V*) ratio is large enough. Hypothermia reduces metabolism in accordance with Arrhenius’ law. The ventilatory response and anapyrexic signaling in small animals (large *SA*:*V* ratio) inhibit the decrease in *P*_a_O_2_ by improving oxygenation and reducing O_2_ consumption, respectively. Isoflurane is known to influence thermoregulation by promoting heat loss mechanisms (a.o., vasodilation). Activating/stimulatory relationships between variables are depicted by arrows, inhibitory relationships by squares.

Several compounds (i.e., hibernation inducing agents, HIAs) have been identified as potential HIT-mimicking agents^12–15^. Hydrogen sulfide gas (H_2_S) was introduced as a lead candidate based on experiments in mice^16,17^. The hypometabolic effects of H_2_S in mice, however, seem to be stooled on a hypoxic mechanism, as recently demonstrated^18^, instead of a HIT-based process. In an experimental setting, exposure of rodents to hypoatmospheric inspiratory oxygen fractions (*F*_i_O_2_) induces a hypometabolic state that physiologically emulates a state of suspended animation as observed in natural hibernators, which is schematically depicted in Fig. 1A. In response to acute hypoxia exposure (*F*_i_O_2_ of 5–10%), mice and small rats lower their *T*_b_ to (deep) hypothermia^19–22^. Hypoxia-induced hypothermia seems to be based on a central Z_tn_-downmodulating mechanism resulting in anapyrexic signaling^2,15,23,24^ and is predicated on the condition that the animal’s surface area (*SA*):volume (*V*) ratio is large enough to allow efficient cooling^2,15^. Activating hypoxia-initiated anapyrexia without the deleterious effects of hypoxia on the organism may hence enable artificial hibernation and a protective metabolic condition.

The body’s primary peripheral oxygen sensing tissues are the carotid chemoreceptors, or carotid bodies (CBs), bilaterally located near the bifurcations of the common carotid arteries^25,26^. The CBs are an essential link in the ventilatory response to hypoxia as they innervate the afferent sinus nerve in response to reduced partial arterial O_2_ pressure (*P*_a_O_2_). This response is referred to as the hypoxic ventilatory response (HVR) and is schematized in Fig. 1B. Acute exposure to 10% *F*_i_O_2_ induces hyperventilation in urethane-anesthetized mice and rats, as well as in conscious rats, as evidenced by an increased respiratory rate and tidal volume^27,28^. Bilateral carotid body resection or denervation in both animal and human studies shows a loss of HVR with immediate hypoventilation and respiratory acidosis^29,30^.

Bilateral denervation of the carotid chemoreceptors reduces ventilation under normoxic conditions and abolishes the hyperpneic HVR (*F*_i_O_2_ = 10%) in urethane-anesthetized mice^27^. Hypoxia exposure in urethane-anesthetized rats deactivates the POAH-regulated, thermogenic brown adipose tissue (BAT), an effect that is abolished in bilateral chemodenervated rats^31^. Altogether, an essential role for the carotid chemoreceptors in hypoxia-induced anapyrexia is suggested. Although a reduction in *P*_a_O_2_ (hypoxemia) emanates from exposure to a hypoxic atmosphere^30^ and a downmodulation of POAH occurs during hypoxemia in rodents^21,23,24^, the link between a reduced *P*_a_O_2_ and consequent Z_tn_-downmodulation has remained unconfirmed.

As the carotid chemoreceptors are essential in the respiratory response to reduced *P*_a_O_2_, we hypothesized that the carotid chemoreceptors fulfill an essential role in hypoxia-induced anapyrexia. In this study the hypothesis is tested by exposing bilateral carotid chemoreceptor-denervated mice to hypoatmospheric *F*_i_O_2_ and comparing the thermographic (i.e., metabolic) effects of hypoxia to those in sham-operated and control mice. Anapyrexic signaling was measured thermographically as described before^15,18,32^ in line with the established strong correlation between rectal temperature and surface temperature (*T*_s_)^33–37^. The induction of a state of suspended animation was additionally measured by automated motion analysis.

## Materials and Methods

### Animals and experimental design

Thirty-three female C57Bl/6 mice with a mean ± SD body weight (BW) of 20.1 ± 1.6 g at the start of the experiment were obtained from Charles River Laboratories (L’Arbresle, France). Mice were given *ad libitum* access to water and food (CRM pellet food, Special Diet Services, Essex, UK) and housed under standard conditions in a temperature- (*T*_a_ = 21.3 ± 0.8 °C) and humidity-controlled room (39.9 ± 4.9%). All animal experiments were approved by the animal ethics committee (protocol # BEX48) of the Academic Medical Center, University of Amsterdam. Animals were treated in compliance with institutional guidelines and the *National Institute of Health Guidelines for the Care and Use of Laboratory Animals* (NIH publication No. 15-8013).

Animals were randomly allocated to one of four experimental groups. Animals in group A (denervation group, N = 8) and group B (sham group, N = 9) were subjected to bilateral CB denervation or sham surgery (incision and mobilization of the carotid arteries), respectively. Animals in both groups were allowed to recover from the anesthesia and surgery during 2 d. Next, the metabolic effects of exposure to a hypoxic atmosphere were determined by thermographic imaging of animals in groups A, B, and C (hypoxia group, N = 8). Animals in group D (normoxia group, N = 8) were continuously exposed to normoxic ambient air.

### Phase I: Anesthesia and surgical procedures

On experimental day 1, animals in the denervation and sham groups received analgesic care by subcutaneous injection of buprenorphine (0.05–0.1 mg/kg BW in 10 mL/kg saline; Reckitt Benckiser, Slough, UK). After approximately 15 min, induction anesthesia was given by inhalation of isoflurane (3.0–4.0%) in mixed room air:O_2_ (1.0:1.0 L∙min^−1^). Isoflurane inhalation anesthesia was maintained at 2.0–3.0%. A temperature probe was placed rectally to monitor the *T*_b_, which was maintained at 36.7–37.3 °C using an infrared lamp and a heating pad.

**Figure 2:**
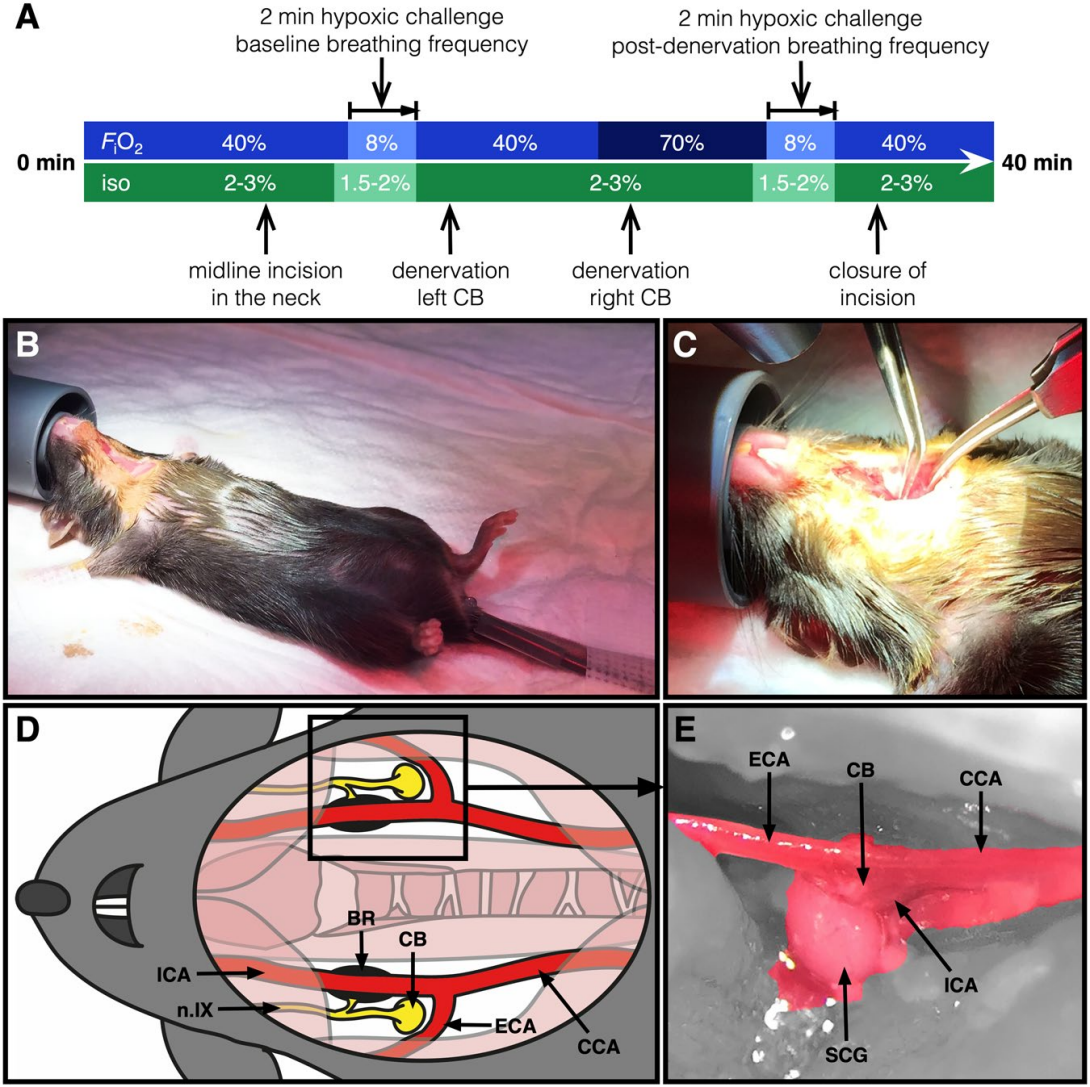
Mouse model of bilateral carotid body denervation. Each depicted procedure is explained in the Materials and Methods section. (**A**) *F*_i_O_2_ and isoflurane (iso) levels and timing during the surgical procedure and HVR testing. In short, (**B**) anesthesia is induced and maintained by subcutaneous buprenorphine injection and isoflurane inhalation. A rectal temperature probe is applied, the animal is fixated, and a midline incision in the neck is made. (**C**) The bifurcation of the carotid artery accessed on each side by blunt preparation. A schematic (**D**) and microscopic anterolateral perioperative image (**E**) of the anatomy of the carotid chemoreceptor area is depicted (15× magnification) showing the carotid body (CB) (after removal of) the superior cervical ganglion (SCG), carotid baroreceptor (BR), glossopharyngeal nerve (n.IX), common carotid artery (CCA), external carotid artery (ECA), and the internal carotid artery (ICA).

For CB denervation, a midline incision in the neck was made in the animals of the denervation and sham groups (surgical procedures are depicted in Fig. 2). Both bifurcations of the common carotid artery were mobilized by blunt dissection (Fig. 2B–E). A baseline HVR test (Fig. 2A) was performed. During the HVR test, isoflurane anesthesia was maintained at 1.5–2.0%. The *F*_i_O_2_ was controlled by mixing O_2_ and nitrogen (N_2_) at ratios of 0.4:0.6 L∙min^−1^ (*F*_i_O_2_ of 40%), 0.08:0.92 L∙min^−1^ (*F*_i_O_2_ of 8% during HVR test), or 0.7:0.3 L∙min^−1^ (*F*_i_O_2_ of 70% during right CB denervation) using dedicated gas valves connected to the gas cylinders. The O_2_ concentration was confirmed prior to the experiment (O_2_ meter, model OdaLog 7000, App-Tek International, Brendale, Australia).

In animals of the denervation group, the left CB (at *F*_i_O_2_ of 40%) and the right CB (at *F*_i_O_2_ of 70%) were denervated by resecting the superior cervical ganglion (SCG) and underlying neural tissue, while sparing surrounding vascular tissue (Fig. 2B–E). The denervated CBs were fixed in formalin (10% buffered formalin solution, Avantor Performance Materials, Deventer, the Netherlands) for histological analysis. In animals of the sham group, the same left and right carotid areas were manipulated but without CB denervation.

After these procedures, another HVR test was performed to confirm successful CB denervation or control manipulation (Fig. 2A). Respiratory rates (RR) were determined by counting the amount of breaths over 15 s at normoxia, and after 50 and 100 s of exposure to an atmosphere comprising an *F*_i_O_2_ of 8%. RR are reported in breaths∙min^−1^. Successful denervation of the CBs was confirmed by the hypoxia-driven increase in RR within 2 min of *F*_i_O_2_ 8% exposure as described before in CB-denervated mice^27^ and confirmed histologically.

### Phase II: Anapyrexia induction procedures

**Figure 3:**
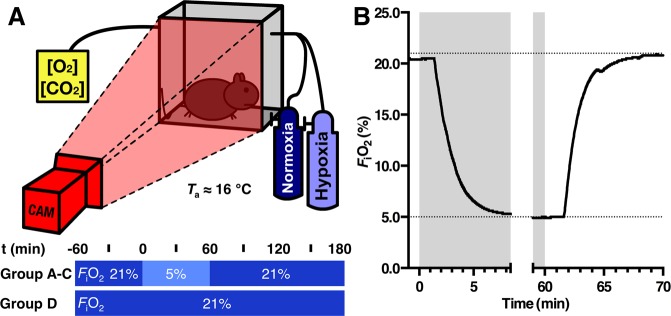
Overview of the anapyrexia induction procedures. (**A**) Schematic overview of the experimental setup used for animal temperature measurements in an airtight cage. A single box is depicted for simplicity of the otherwise 9 boxes arranged in a 3 × 3 array. A thermographic camera (CAM) was employed to record temperature. *F*_i_O_2_ was controlled by the gas mixture connected to the gas inlet of the cage, whereas O_2_ and CO_2_ concentrations were measured at the outlet. An adapted version of this drawing was published before under CC BY license (https://creativecommons.org/licenses/by/4.0) by our group^18^. (**B**) Representative *F*_i_O_2_ dynamics in mice during the atmosphere change from normoxia to hypoxia (-1–8.5 min) and back to normoxia (59–70 min).

On experimental day 3, surgically treated animals (denervation and sham groups) as well as non-treated animals (hypoxia control and normoxia groups) were placed in the experimental setup and subjected to the experimental conditions as depicted in Fig. 3. From −60 up to 0 min animals of all groups were exposed to the same normoxic atmosphere of ambient air (*F*_i_O_2_ of 21%, O_2_ meter, model OdaLog 7000, App-Tek International). From 0 to 60 min, animals in the denervation-, sham-, and hypoxia control groups were exposed to a hypoxic atmosphere (*F*_i_O_2_ = 5%, *F*_i_N_2_ = 95%; Linde Gas, The Linde Group, Munich, Germany), which was followed by 120 min of recovery by switching back to the normoxic atmosphere. In contrast, exposure of the normoxia group to the normoxic atmosphere was continued from 0 to 180 min. The *T*_s_ and locomotor activity were determined by thermographic imaging (ThermaCAM SC2000, FLIR Systems, Wilsonville, OR). Animals were filmed every 15 min for 120 s and, in addition, from t = 0 up to t = 9 min in the denervation-, sham-, and hypoxia control groups. Thermographic images were obtained at a frame rate of 3 FPS and saved as grayscale bitmap files using ThermaCAM Researcher 2001 software (FLIR Systems). As the experimental setup consisted of nine atmosphere-controlled boxes configured in a 3 × 3 matrix^18^, a maximum of nine animals were filmed simultaneously.

At 180 min, animals were sacrificed by pentobarbital injection (100 mg/kg BW i.p.; AST Farma, Oudewater, the Netherlands). Immediately after sacrifice, cardiac mixed arterial/venous blood samples of approximately 0.5 mL were collected by cardiac puncture and transferred to heparin-flushed 1 mL syringes (5,000 IE/mL heparin with minimal residual volume, LEO Pharma, Amsterdam, the Netherlands). The CBs of animals in the sham-, hypoxia control-, and normoxia groups were harvested from the total carotid bifurcation area and formalin-fixed for histological analysis.

To ascertain sufficient inflow rates and prevent CO_2_ accumulation, CO_2_ concentrations were determined at the gas outlet (<600 ppm; CO_2_ Meter, Ormond Beach, FL). During the entire experiment, the *T*_a_ in the cages was maintained at 15.8 ± 0.4 °C via air conditioning in the operating room, as confirmed by the CO_2_ meter placed at the gas outlet. For purposes of *F*_i_O_2_ accuracy, an O_2_ concentration meter (Fig. 3B; model OdaLog 7000, App-Tek International) was also connected to the outlet.

### Analysis of thermographic images

Analysis of temperature and motion was performed with dedicated software (MatLab 2013a, MathWorks, Natick, MA) by loading the grayscale bitmap files. For each animal, the individual average minimum and maximum temperature of all frames per time point were determined. The 15-min interval 2-min time points in all groups and the 1–9 min interval 1-min time points in the denervation-, sham-, and hypoxia control groups comprised 360 and 180 frames, respectively. Subsequently, the mean *T*_s_ was calculated per time point per group.

For motion analysis, the same thermographic images were used as for temperature analysis. Fluctuations in grayscale pixel intensity of consecutive images were determined and averaged per time point. A pixel was considered to reflect animal movement when the grayscale intensity difference exceeded 7 on a scale of 0 to 255, accounting for the background scatter. This cut-off was determined in pilot analyses of 11 120-s video sequences. Values were expressed as the mean ± SEM amount of pixels with ‘motion’ per group per time point.

### Histological and biochemical analyses

Formalin-fixed denervation tissue from animals of the denervation group as well as formalin-stored carotid bifurcations of all other mice were dehydrated in graded steps of ethanol and xylene and embedded in paraffin. Five-μm sections were stained with hematoxylin and eosin (HE). To histologically confirm successful CB denervation, biopsies were examined for the presence of SCG tissue. In addition, carotid bifurcation specimens of the denervation and sham groups were analyzed for the absence and presence of both SCG and CB tissue, respectively.

Blood gas analysis of arterial/venous mixed blood samples was performed using an ABL80 FLEX gas analyzer (Radiometer, Brønshøj, Denmark).

### Statistical analysis

Specific statistical analyses were performed in MatLab 2013a. Of each group, homogeneity of variance was tested using the Bartlett’s test, which was assumed for *P* ≥ 0.05. The intergroup differences of RR, *T*_s_, and locomotor activity were compared per time point using a one-way ANOVA in case of homogeneity or a Kruskal-Wallis test in case of heterogeneity, followed by a Tukey’s range test or Dunn’s test, respectively.

The RRs were normalized to normoxia (control) values of the paired hypoxic challenge per animal. Repeated measurement (RM)-ANOVA was performed in SPSS (IBM Corporation, Armonk, NY) to determine the effects of CB denervation and hypoxia exposure on the RR. As evidenced by Mauchly’s test of sphericity, sphericity was not violated in any of the RM-ANOVA tests. *P*-values less than 0.05 were considered significant. All values were presented as mean ± SEM unless noted otherwise.

## Results

### The ventilatory response is abrogated in carotid body-denervated mice

**Figure 4:**
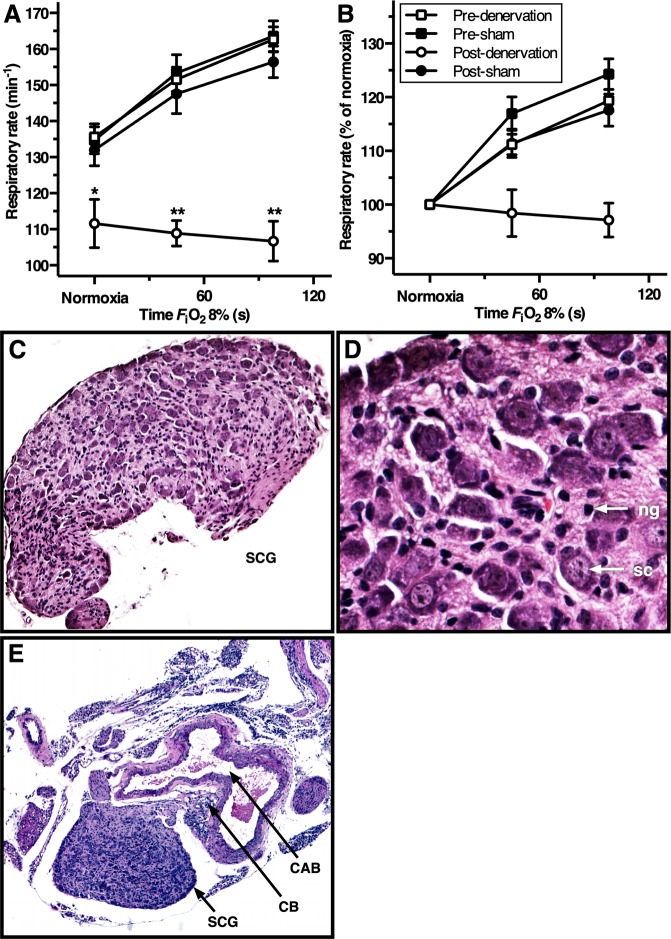
HVR and histological confirmation of CB denervation. (**A**) Mean ± SEM respiratory rate (RR) and (**B**) mean ± SEM RR normalized to normoxia and expressed as a function of hypoxia time (*F*_i_O_2_ of 8%, from 0–100 s) for the animals in the denervation and sham group before and after the surgical procedure. Statistically significant differences per time point of hypoxia exposure were found in post-denervation vs. others (*P < 0.01; **P < 0.001). One-way repeated measurements ANOVA on normoxia-normalized RR values of the denervation group yielded a procedure effect, time effect, and interaction between both (*P* < 0.001, *P* < 0.05 and *P* < 0.01, respectively), whereas the same test in the sham group only found a time effect (*P* < 0.001). In the sham group a procedure effect and interaction were absent (*P* = 0.537 and *P* = 0.811, respectively). (**C**–**E**) Hematoxylin-eosin (HE)-stained histology images (100×, 400×, and 50× magnification, respectively) showing the SCG neuroglia cells (ng), satellite cells (sc), CB, and the carotid arterial bifurcation (CAB). The SCG depicted in panels C and D was obtained from the denervation group, whereas the tissue depicted in panel E was obtained from an animal from the sham group.

To functionally confirm complete bilateral surgical denervation of the CBs, the HVR was determined by measurement of the RR before and after CB denervation or sham operation during hypoxia (*F*_i_O_2_ = 8%). In line with expectations, the RR under normoxic conditions was 18% lower in CB-denervated animals (*P* < 0.01) in contrast to a 4% drop in the sham-operated animals. CB-denervated animals exposed to hypoxic conditions for 120 s reduced their RR by 4%, whereas sham-operated animals increased their RR by 20% following 120 s of hypoxia exposure, which is consistent with pre-operating procedure values (Fig. 4A,B). The RR at normoxic *F*_i_O_2_ and the HVR were significantly reduced after CB denervation, indicating CB absence in animals of the denervation group. In contrast, the animals of the sham group retained functionally intact CBs and normophysiological responsiveness.

### Rewarming after hypoxia-induced hypothermia is abrogated in mice lacking carotid bodies

**Figure 5:**
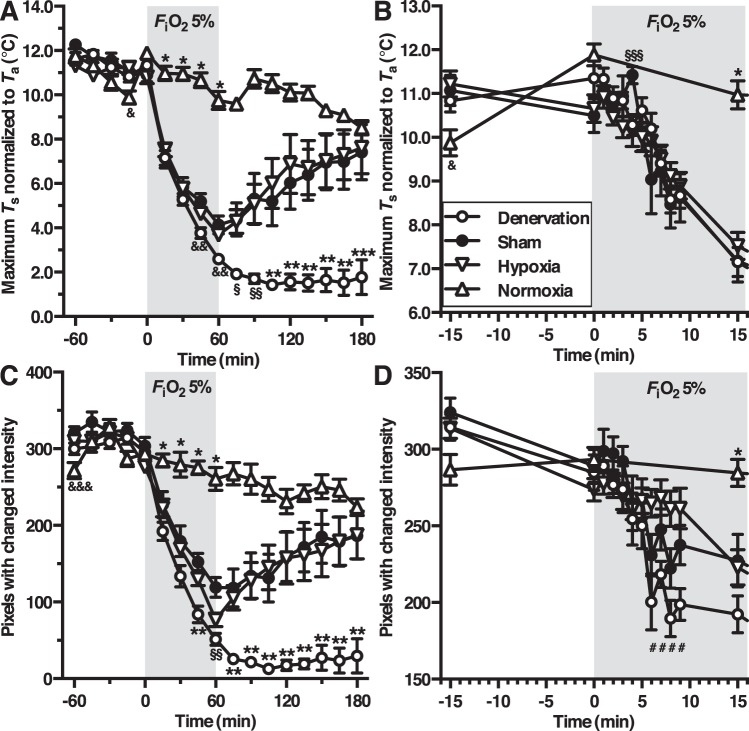
Body temperature (**A**,**B**) and locomotor activity (**C**,**D**) during hypoxia-induced anapyrexia. The legend is provided in panel B. Group and statistical test indices are depicted as following: normoxia vs. other groups (*P < 0.001), denervation vs. other groups (**P < 0.001, or ***P < 0.01), normoxia vs. hypoxia (^&^*P* < 0.05), normoxia vs. sham and denervation (^§^*P* < 0.001), denervation vs. sham and normoxia (^§§^*P* < 0.001), sham vs. hypoxia (^§§§^*P* < 0.05), sham vs. denervation (^&&^ *P* < 0.001), normoxia vs. sham and hypoxia (^&&&^ *P* < 0.01), and denervation vs. hypoxia (^#^*P* < 0.05).

To assess the animals’ *T*_b_ kinetics during changing *F*_i_O_2_ conditions, thermographic imaging was performed at 15-min intervals in all groups (Fig. 5A). Except for a small transient drop in the hypoxia group at t = −15 min (*P* < 0.05), all baseline (t = −60–0 min) maximum *T*_s_ values did not differ between the groups. After exposure to hypoxia (*F*_i_O_2_ = 5%), the maximum *T*_s_ dropped immediately in animals of the denervation-, sham-, and hypoxia control groups compared to the normoxia group (*F*_i_O_2_ 21%). The *T*_s_ decline persisted during the total time of hypoxia exposure (*P* < 0.001). Animals in the sham and hypoxia control group decreased their maximum *T*_s_ to 4.1 ± 0.4 °C and 3.7 ± 0.2 °C above *T*_a_, respectively, whereas CB-denervated animals lowered their *T*_s_ to 2.6 ± 0.2 °C above *T*_a_ at the end of the exposure period.

At t = 60 min, the atmosphere in the denervation-, sham-, and hypoxia control group was reverted to normoxia (*F*_i_O_2_ = 21%) and the animals were allowed to recover from hypoxia exposure. The animals in the sham and hypoxia control groups raised their maximum *T*_s_ within 120 min (7.4 ± 1.3 °C and 7.6 ± 1.2 °C, respectively) to normoxia control levels (8.5 ± 0.3 °C) relative to *T*_a_. The CB-denervated animals were unable to rewarm to normoxia control levels, as evidenced by a maximum *T*_s_ rise of 1.8 ± 0.8 °C above *T*_a_ at t = 180 min. In fact, after switching back to a normoxic atmosphere, the maximum *T*_s_ of CB-denervated animals dropped even further to 1.4 ± 0.2 °C above *T*_a_ during the first 105 min, veering significantly from the temperature patterns exhibited by animals in the sham-, hypoxia control-, and normoxia groups (*P* < 0.001).

To examine *T*_b_ reduction during the transition between normoxic and hypoxic air (Figs. 3B), 1 min average maximum *T*_s_ of the denervation-, sham-, and hypoxia control groups were analyzed between t = 0 min and 9 min (Fig. 5B). Exposure to hypoxia (*F*_i_O_2_ = 5%) induced an immediate cooling response in all hypoxia-exposed animals without any intergroup differences other than a small increase of the maximum *T*_s_ at t = 4 min in sham-operated animals (group B, *P* < 0.05).

### Restoration of locomotor activity after hypoxia-mediated hypothermia is abrogated in carotid body-denervated mice

To determine the animals’ locomotor activity (where a hypometabolic state corresponds to an absence of locomotor activity), grayscale pixel intensity differences were determined on the same thermographic images as used in the aforementioned temperature analyses. The time point average amount of pixels with movement of baseline (t = −60–0 min) did not differ between groups except for a slight reduction in the amount of pixels with movement at t = −60 min in the normoxia group (*P* < 0.01, Fig. 5C). During the total time of hypoxia exposure, animals of the denervation-, sham-, and hypoxia control groups significantly decreased their movement compared to animals in normoxia group (*P* < 0.001). The decrease in movement due to hypoxia exposure was significantly more prominent in the CB-denervation group at t = 45 min compared to the other groups (*P* < 0.001), as well as at t = 60 min compared to the sham and normoxia group (*P* < 0.001). After cessation of hypoxia exposure at t = 60 min, the movement in denervation group declined further from 51 ± 8 pixels end-exposure (t = 60 min) to 13 ± 3 pixels at t = 105 min and 30 ± 23 at t = 180 min (all *P* < 0.001), whereas both the sham and hypoxia control group returned to normoxia group levels within 120 min of recovery (186 ± 31, 187 ± 31, and 224 ± 11 pixels at t = 180 min, respectively).

Similar to the temperature analyses, locomotor activity from animals in the denervation-, sham-, and hypoxia control groups were determined at 1-min temporal resolution from t = 0–9 min. Exposure to an *F*_i_O_2_ of 5% induced a prompt reduction in movement in all hypoxia-exposed groups, which was more profound in the CB denervation group compared to hypoxia control group between t = 6–9 min (*P* < 0.05, Fig. 5D). As mentioned before, this difference in movement of animals in the denervation and hypoxia control groups was absent at t = 15 min, at which point the movement of animals in the hypoxia control group had declined to levels of the denervation- and sham groups.

### Animals that did not respond to hypoxia lacked an intact carotid body signaling machinery

To confirm successful carotid chemoreceptor denervation, the denervation tissue of all animals in the denervation group was stored after surgery and analyzed by light microscopy. The SCG tissue was typified by the presence of nerve cell bodies and satellite cells and the CB tissue by glomus type I cells and glia cells (Fig. 4C,D). All denervation specimens obtained from the denervation group were characterized on the basis of this SCG phenotype (18 out of 18 CBs from 9 animals).

To ascertain complete denervation in the denervation group as well as the presence of CBs in all other groups, the total carotid bifurcation with surrounding tissues was harvested after animal sacrifice (N = 3 from hypoxia control and normoxia groups; Fig. 4E).

### Acidosis is aggravated in mice lacking carotid bodies

Blood gas analysis was performed to determine oxygenation, acid-base balance, and circulatory electrolytes. Due to premature death of one animal in the denervation group, blood gas analysis was performed on 8 animals in the denervation group. The data are presented in Table 1.

**Table 1:** Blood gas analysis. Blood gas analysis performed on mixed arterial-venous blood samples obtained by cardiac puncture immediately after sacrifice.

Parameter	Denervation (group A)	Sham (group B)	Hypoxia (group C)	Normoxia (group D)	*P*-value
	(N = 8)	(N = 9)	(N = 8)	(N = 8)	
***Oxygenation***					
*P*O_2_ (mmHg)	**107 **±** 18**^**&**^	80 ± 14	**49 **±** 5**	62 ± 7	0.005
*s*O_2_ (%)	**90 **±** 2**	**84 **±** 5**	**65 **± 6**	82 ± 4	0.011
*ct*O_2_ (vol-%)	**21 **± 1***	**15 **±** 1**	**13 **±** 1**	**15 **±** 1**	<0.001
Hct (%)	**52 **± 1*	**40 **±** 2**	43 ± 1	**40 **±** 1**	<0.001
*ct*Hb (g·dL^−1^)	**17 **± 0^***^	**13 **±** 1**	**14 **±** 0**	**13 **±** 0**	<0.001
***Acid-base homeostasis***					
pH	**7**.**01 **±** 0**.03*	**7**.**21 **±** 0**.**02**	**7**.**11 **±** 0**.03*	**7**.**24 **±** 0**.**01**	<0.001
*P*CO_2_ (mmHg)	**84 **± 5*	**51 **±** 5**	70 ± 5	**59 **±** 1**	0.003
*ct*CO_2_ (mmol·L^−1^)	**19 **±** 1**	**18 **±** 1**	20 ± 1	**23 **± 1^**^	0.001
*c*HCO_3_^−^ (mmol·L^−1^)	**20 **±** 1**	**19 **±** 1**	**21 **±** 1**	**24 **± 1^***^	<0.001
BE (mmol·L^−1^)	**−15 **± 1^***^	**−9 **±** 1**	**−10 **±** 1**	**−4 **± 1^***^	<0.001
Anion gap (mmol·L^−1^)	24 ± 1	25 ± 1	**26 **±** 1**	**23 **±** 1**^**&**^	0.012
***Electrolytes***					
*c*Na^+^ (mmol·L^−1^)	150 ± 1	150 ± 1	152 ± 1	152 ± 0	0.104
*c*K^+^ (mmol·L^−1^)	**4**.**0 **±** 0**.**2**^**&&**^	3.7 ± 0.1	**3**.**4 **±** 0**.**1**	**3**.**4 **±** 0**.**1**	0.015
*c*Ca^2+^ (mmol·L^−1^)	1.2 ± 0.1	1.1 ± 0.0	1.0 ± 0.1	1.1 ± 0.1	0.191
*c*Cl^−^ (mmol·L^−1^)	110 ± 1	111 ± 1	109 ± 1	108 ± 1	0.076

Statistically significant * vs. sham and normoxia; **vs. denervation and sham; ***vs. all other groups; ^&^vs. the hypoxia group; ^&&^vs. hypoxia and normoxia. All statistically significant values are marked in boldface.

Abbreviations: Partial oxygen pressure (*P*O_2_), oxygen saturation (*s*O_2_), oxygen content (*ct*O_2_), hematocrit (Hct), hemoglobin content (*ct*Hb), partial carbon dioxide pressure (*P*CO_2_), carbon dioxide content (*ct*CO_2_), bicarbonate concentration (*c*HCO_3_^−^), base excess (BE), sodium concentration (*c*Na^+^), potassium concentration (*c*K^+^), calcium concentration (*c*Ca^2+^), chloride concentration (*c*Cl^−^).

Given the pH- and *P*_a_CO_2_ values in the normoxia group, animals in all groups exhibited procedure-related acute respiratory acidosis. The normophysiological pH- and *P*_a_CO_2_ values are 7.35–7.45 and 35–45 mmHg, respectively. In addition, animals in all hypoxia-exposed groups suffered metabolic acidosis, as further corroborated by the base excess (BE) levels. The degree of acidosis in the denervation group was significantly greater than in the sham- and normoxia groups, whereas a trend was observed relative to the hypoxia control group. This was anticipated inasmuch as the CB-null animals could not engage an HVR and hence acidified more profusely. The lower BE in the denervation group compared to all other groups, as well as the sham and hypoxia control groups compared to the normoxia group, suggests more respiratory compensation in CB-denervated animals as well as the animals of the sham and hypoxia control group, respectively, most likely via unimpaired pH-sensitive chemoreceptors in the central nervous system^38–41^.

In terms of oxygenation, the *P*_a_O_2_ level in denervation group exhibited an augmentative trend compared to that in the sham group and the normoxia group (34% and 73%, respectively) and was significantly higher relative to the hypoxia control group (118%). Likewise, the hematocrit (Hct) and total hemoglobin concentration (*c*tHb) in denervation group were significantly elevated compared the other groups, except for the Hct of the denervation group versus the hypoxia control group.

No notable deviations in the electrolyte levels were observed, except for a minimal increase in potassium (K^+^) concentration in the denervation group compared to the hypoxia control- and normoxia groups (approximately 19%), which can be explained by the severe acidosis in the denervation group^42,43^. This features a common clinical phenomenon that entails an intracellular shift of H^+^ ions in acidosis followed by an extracellular shift of K^+^ ions to maintain electroneutrality.

## Discussion

Hibernation in humans could be beneficial in medicine, sports, and aviation^2^. Despite decades of research, a true HIA for the artificial induction of hibernation in humans is yet to be unraveled^5^. Hypoxia exposure induces a protective hibernation-like state (i.e., anapyrexia) in rodents (Fig. 1A)^18,23,24^, which could be the key to the implementation of a hibernation-like state in the clinical setting^2^. As hypoxia itself has deleterious effects (i.e., ischemia and reperfusion injury)^6,8^, mimicking hypoxia-mediated anapyrexic signaling without suffocation and tissue injury is quintessential. Given that CBs are instrumental in the HVR^27^, the role of the carotid chemoreceptors in hypoxia-induced anapyrexia was explored in this mouse study (Fig. 1B). The CB denervation experiments revealed that CBs indeed play a role in anapyrexic effects under conditions of low oxygen. However, CBs do not affect the induction of anapyrexia but mainly impair the recovery from hypoxia during subsequent normoxia.

It should be noted that, in addition to the role of CBs in hypoxemic hypoxia-mediated anapyrexia, other forms of hypoxia may induce anapyrexia. Carotid chemoreceptors are primarily sensitive to differences in unbound, dissolved O_2_ in the arterial blood (i.e., *P*_a_O_2_). Anemic hypoxia (reduction in O_2_ content) and circulatory hypoxia (reduction in *D*O_2_) could trigger anapyrexia via the aortic chemoreceptors and baroreceptors, respectively^44–46^. Here, the anapyrexic effects of hypoxemic hypoxia were investigated in the context of carotid chemoreceptors because, from a practical view, (1) the extent of *P*_a_O_2_ reduction can be controlled more easily than other forms of hypoxia, and (2) the surgical approach of the CBs is less invasive compared to aortic chemoreceptor resection and hence more suitable in a model that entailed awaking from mild anesthesia.

### Technical considerations regarding our animal model: CB ablation and anesthesia

The carotid chemoreceptors have been the subject of study for decennia and have gained interest from several research groups, who primarily have focused on the intrinsic pathways of hypoxia sensing that results in a ventilatory response^25,47,48^. Several methods to eliminate CB function in animals have been published. Surgical methods comprise complete resection^49,50^ or glossopharyngeal nerve denervation^27,31,51^, whereas selective interference with CB function has been performed pharmacologically^52^ and genetically^53,54^. In this study, bilateral surgical denervation was performed to ensure complete loss of CB function. During the denervation procedure, SCG tissue was bilaterally identified and resected with precision in order to prevent damage to surrounding vascular tissue such as the carotid baroreceptors (Figs 2C–E and 4). Although removal of the SCG results in loss of sympathetic function of head and neck, its removal does not interfere with thermoeffective tissue function such brown adipose tissue (BAT), as BAT-thermogenesis is innervated by the thoracic stellate ganglion^55^. Confirmation of complete functional resection by HVR testing was in accordance with experiments in CB-denervated mice under urethane anesthesia^27^.

In previous experiments comprising CB surgery in rodents, urethane anesthesia was used to prevent the ventilation suppressing effects of inhalation anesthetics such as isoflurane. Isoflurane is known for its dose-dependent inhibition of the ventilatory regions of the brain^56^, which prevents the accurate determination of CB function. However, given the need for an anesthetic from which mice could awake rapidly, low dose isoflurane anesthesia (1.5–2.0%) was utilized during surgical procedures (i.a., CB function tests). To prevent bias caused by thermoregulatory effects of isoflurane, hypoxia-exposed metabolism experiments were performed two days after isoflurane anesthesia^57,58^.

### Cooling rate and depth following hypoxia-induced hypothermia induction are dependent on the extent of the T_s_ − T_a_ mismatch

In accordance with previous literature^19,21,22^, our study showed that mice with intact carotid chemoreceptor function exposed to a hypoatmospheric *F*_i_O_2_ of 5% (i.e., sham- and hypoxia group) drop their *T*_b_ to a state of deep hypothermia. The effect size (cooling rate as well as the extent of the cooling response) in all hypoxia-exposed groups was greater at a *T*_a_ of 16 °C than that observed in our earlier experiments performed at a *T*_a_ of 21 °C (Fig. 1A and^18^). Accordingly, the rate and depth of the hypothermic response to hypoxia-mediated Z_tn_-downmodulation seems to be *T*_a_-dependent and attests to the passive cooling principle in the context of a facilitatory *SA*:*V* ratio (Fig. 1B). That is why mice (high *SA*:*V* ratio) exhibit *T*_s_ ≈ *T*_a_ accommodation, and do so more profoundly when *T*_a_ ≪*T*_s_, while pigs (low *SA*:*V* ratio) do not^59^, even when exposed to ice-cold temperatures and potent, intravenously administered HIAs (manuscript in preparation).

### Hypoxia-induced hypothermia is likely governed by central and peripheral mechanisms

However, elimination of carotid chemoreceptor O_2_ sensing by bilateral CB denervation did not diminish the acute hypothermic response to hypoxia. In fact, CB-denervated mice in the abovementioned experiments reduced their *T*_s_ upon exposure to an *F*_i_O_2_ of 5%, even to a greater extent than sham-operated animals. As posited previously by our group^2,18^, central nervous system-regulated hypothermia (i.e., POAH downmodulation) will lead to a reduction in metabolic rate due to the Arrhenius effect (*T*_b_ ↓ ↔ metabolic rate ↓)^3,4^. Inasmuch as this central mechanism (in this case driven by hypoxemic hypoxia owing to exposure to subatmospheric O_2_ levels) is absent in CB-denervated mice, a peripheral mechanism is more likely to be responsible for the observed hypometabolic effects. Note that mice in group D (normoxia) exhibited homeothermy, while normophysiological *T*_s_ regulation was compromised in the hypoxia groups. Since CB-ablated mice cannot sense hypoxia and no hypoxia sensing mechanisms resulting in HVR are known other than CB sensing^30^, these animals were expected to adopt a response that paralleled the one in mice of the normoxia group (i.e., no cooling). This was, however, not the case.

We would like to offer a possible explanation for these unexpected observations. Alongside the *T*_b_-dependency, the Arrhenius equation takes into account substrate dependency^2^. In response to reduced substrate (O_2_) availability, the metabolism shifts from oxidative phosphorylation to anaerobic respiration/glycolysis. This metabolic shift is accompanied by augmented lactate production and consequently metabolic acidosis, which is generally compensated by the respiratory system through central chemoreceptor-mediated hyperventilation and an increase in CO_2_ expiration^38–41^. Under conditions of high metabolic demand (i.e., *T*_a_ ≪Z_tn_ such as during the first 15 min of gas exposure in our experiments), a shift to anaerobic metabolism could result in lower heat production (i.a., reduction in the oxygen-dependent BAT thermogenesis^60^) and therefore in forced hypothermia^2^. Forced hypothermia entails a state where the *T*_b_ is driven below the Z_tn_, as would occur during acute exposure to low *T*_a_ or exposure to drugs that impair thermogenesis. In the absence of POAH-downmodulation, the progressive acidosis and inability to maintain a sufficient metabolic rate in CB-denervated animals may have caused the temperature drop. Although hypoxia-induced forced hypothermia differs fundamentally from hypoxia-induced anapyrexia, both culminate in cooling.

### CB-denervated animals likely cool down as a result of forced hypothermia instead of anapyrexia

The blood gas data lend credence to the above. All animals exhibited acidosis (Table 1), which can (partly) be explained by the effects of pentobarbital-induced respiratory depression^61,62^ prior to harvesting of the blood samples. Accordingly, animals in all groups suffered from acute respiratory acidosis as evidenced by hypercapnia. On top of the respiratory acidosis in all hypoxia-exposed groups, metabolic acidosis was observed as evidenced by a decreased BE and HCO_3_^–^, which was most prominent in the CB denervation group. This suggests CB-denervated animals to initially suffer from substrate deficit-induced forced hypothermia without the POAH-downmodulating component, whereas animals in the sham and hypoxia control groups primarily dropped their *T*_b_ in consequence to POAH-downmodulation and hypoxemic hypoxia. As POAH downmodulation ceases thermogenic processes such as BAT thermogenesis and shivering^24,60,63^, hypoxia exposure in the sham and hypoxia control groups immediately led to a reduction in *V*O_2_ and an acute, hypoxemic hypoxia-induced reduction in *D*O_2_ (demand and supply matching). Contrarily, due to a lack of intracellular oxygen (i.e., *D*O_2_/*V*O_2_-mismatch) in CB-denervated animals, oxidative phosphorylation was switched to anaerobic glycolysis in an attempt to maintain a sufficient metabolic rate to sustain normothermia, which is accompanied by excessive lactic acid production and metabolic acidosis (Pasteur effect)^32,64^.

The oxygenation data further support our hypothesis. Metabolic acidosis increases ventilation via the unimpaired pH-sensitive central chemoreceptors in an attempt to resolve the acidosis^38–41^. This response is accompanied by increased oxygen ventilation and hence an increased *P*_a_O_2_, *S*_a_O_2_, and *C*_a_O_2_. Given that the blood samples were obtained after 2 hours of recovery at normoatmospheric *F*_i_O_2_, the augmented *C*_a_O_2_ during recovery from hypoxemic metabolic acidosis suggests the manifestation of forced hypothermia in animals lacking carotid chemoreceptor function.

The putative fundamental difference between forced hypothermia and anapyrexia is the absence of hypoxia-induced, central-mediated cessation of thermogenesis without carotid chemoreceptor function^31^. At least, this difference was formulated based on experiments in rats. Compared to rats with intact carotid sinus nerves, CB-denervated rats under urethane anesthesia lack the autonomic cooling response to hypoxia-mediated reduction in BAT activity (i.e., cessation of BAT sympathetic nerve activity and the consequent decrease in BAT temperature). Consistently, CB-denervated rats also exhibit a hypothermic response to *F*_i_O_2_ 12% exposure, which is characterized by an increased thermogenic shivering intensity compared to CB-intact rats^29^. However, due to decreased oxygen sensing in the hypoxia-subjected, CB-denervated rats, ventilation (RR and tidal volume) increases at a lower rate than in wild type rats, which explains a more prominent *V*O_2_ reduction in the animals lacking CBs. Although a reduction in *V*O_2_ as part of hypometabolism is a hallmark of (artificial) hibernation, the *V*O_2_ reduction should coincide with hypothermia in accordance with the Arrhenius’ equitation^2–4^ to attain protection against ischemia/reperfusion injury. This is the case in anapyrexia^24,65^.

In the blood gas data of the CB-denervated mice the hemoglobin concentration and hematocrit levels are remarkably higher than those of animals in the other groups. As no significant bleeding was observed throughout the experiment in all groups, the most logical explanation is hemoconcentration due to edema, altered renal function, or dehydration. The increase in hemoglobin concentration and hematocrit cannot be ascribed to a hematopoietic origin given the short experimental time frame. At this stage an irrefutable reason for the aberrant hemodynamic variables cannot be established based on the available data.

The blood gas data should be interpreted carefully as blood samples were collected directly after pentobarbital administration. Ideally, blood sampling should be performed during hypoxia exposure as well as during normoxic recovery in the absence of pentobarbital-induced respiratory effects^61,62^. However, blood sampling is invasive inasmuch as it requires anesthetics. Consequently, noninvasive thermal imaging was prioritized in this study as a barometer for metabolism to preclude metabolism-skewing effects of anesthetics.

### Towards the use of a true HIA

In a search for a pharmacological agent that mimics hypoxia-mediated CB activation and anapyrexia under normoatmospheric *P*_a_O_2_s, the presumed HIA H_2_S has been considered^59^. From a mechanistic point-of-view, endogenous H_2_S has an essential role in CB-O_2_ sensing, which can be simulated by exogenous H_2_S in *ex vivo* experiments^25,52,54,66^. The use of exogenous H_2_S gas, or its water-soluble analog NaSH, might be promising for CB activation at normoatmospheric *P*_a_O_2_s. This approach was advocated by the alleged hypothermic potential of exogenous H_2_S in mice^16,17^. However, we recently demonstrated that exogenous H_2_S-induced hypothermia is absent in normoxic mice (*F*_i_O_2_ 21%) and is based on its cumulative effects with mild hypoxia (*F*_i_O_2_ 17%)^18^. Based on the findings in this study, pharmacologically targeting the CBs to modulate the CB-POAH signaling axis may not qualify as a top-tier strategy for the induction of anapyrexia, given the marginal role of CBs in the induction process. Other HIAs that do not necessarily play in on the CB-POAH signaling axis should therefore be prioritized. An extensive summary of potential HIAs can be found in Dirkes *et al*.^15^.

### Concluding remarks

The CBs play a role in hypoxia-mediated anapyrexia and hypometabolism in mice that is mainly evident in the restorative phase rather than the inductive phase following a hypoxic insult. Although elimination of carotid chemoreceptor function by bilateral surgical denervation of the CBs does not prevent hypothermia upon hypoxia exposure, CB-denervated mice were unable to recover from exposure to hypoxia in terms of temperature, breathing, and movement. Consequently, targeting the CB-POAH signaling axis to induce anapyrexia without detrimental hypoxia exposure may have limited efficacy for clinical purposes.
